# Effects of dietary sclareol supplementation in aged broiler breeders on production performance, egg quality, antioxidant capacity, and gut microbiota

**DOI:** 10.1016/j.psj.2025.106317

**Published:** 2025-12-19

**Authors:** Gang Shu, Binghua Zhou, Yang Wang, Zhengkun Wu, Haohuan Li, Funeng Xu, Wei Zhang, Hualin Fu, Lizi Yin, Felix Kwame Amever, Juchun Lin, Yilei Zheng, Xiaoling Zhao

**Affiliations:** a Department of Veterinary Medicine, Sichuan Agricultural University, Chengdu 611130, PR China; b Center for Veterinary Sciences, Zhejiang University, Hangzhou 310058, PR China; c Key Laboratory of Livestock and Poultry Multi-omics, Ministry of Agriculture and Rural Affairs, College of Animal Science and Technology (Institute of Animal Geneticsand Breeding), Sichuan Agricultural University, PR China

**Keywords:** Sclareol, Aged, Broiler Breeder, Antioxidant, Microbiota

## Abstract

This study evaluated the effects of sclareol on production performance, serum antioxidant indices, and gut health in late-laying Tianfu broiler breeders. Six hundred 42-week-old breeders were randomly divided into five groups: basal diet (CON), basal diet with 250 mg/kg oregano thymol (ORE), or basal diet with 200 (LSCL), 400 (MSCL), or 800 mg/kg sclareol (HSCL). Each group included eight replicates of 15 birds. Performance parameters were recorded weekly. On days 42 and 84, samples were collected to assess antioxidant capacity, digestive enzyme activity, and intestinal morphology.

Compared to CON, the MSCL group exhibited a 6.3 % higher egg production rate (*P* < 0.05) and significantly lower cracked and deformed egg rates by 32.1 % and 28.5 % (*P* < 0.05) during days 42–84. Both MSCL and HSCL groups showed increased Total Antioxidant Capacity (T-AOC) and Total Superoxide Dismutase (T-SOD) activities (*P* < 0.05) and an 18.6 % reduction in Malondialdehyde (MDA) content (*P* < 0.01). The MSCL group demonstrated a 22.4 % increase in duodenal villus height and an 18.6 % higher villus-to-crypt ratio (*P* < 0.05), plus a 27.3 % greater jejunal lipase activity (*P* < 0.05). Ileal T-SOD activity increased (*P* < 0.05), and MDA content decreased (*P* < 0.01) in MSCL. FATP4 expression was upregulated in duodenum and jejunum, while SREBF-1 expression increased in jejunum and ileum (*P* < 0.05). Total fatty acid contents in eggs peaked in MSCL (*P* < 0.05). Sclareol supplementation improved intestinal morphology, nutrient digestibility, and antioxidant capacity, enhancing breeder performance, with 400 mg/kg providing optimal benefits.

## Introduction

With the aging of poultry, intestinal function undergoes a multidimensional decline, compromising the chemical, mechanical, and microbial barriers. This systemic deterioration forms the core physiological basis for reduced production performance. Aging impairs the gut's multifaceted barriers including chemical, mechanical and microbial, directly reducing poultry performance.

Compared to younger hens (195, 340 days), 525-day-old hens show reduced activities of key enzymes (sucrase, maltase, trypsin, amylase, lipase) at multiple sites (Gu et al., 2021). Beyond decreased secretion, aging may weaken enzyme-substrate binding (Yamamoto et al., 2014). Critically, reduced mucin production can lead to the leakage of digestive enzymes from the lumen into the lamina propria and systemic circulation, causing local damage and potential accumulation in distal organs like the heart, liver, and brain (DeLano and Schmid-Schönbein, 2024). The intestinal physical barrier is severely compromised. Age-related molecular changes in crypt stem cells impair epithelial renewal (Martin et al., 1998), driven by oxidative stress (Moorefield et al., 2017). Altered villus architecture and increased serum d-lactate indicate heightened permeability (Gu et al., 2021). In aged models, tight junction protein expression (ZO-1, Occludin, JAM-A) decreases while Claudin-2 increases (Wang et al., 2019). Furthermore, aging disrupts microbial balance. The abundance of microbiota involved in beneficial metabolic functions declines with age (Shi et al., 2024). In late lay, high-producing hens exhibit lower diversity but higher *Lactobacillus* abundance (Wang et al., 2020). This dysbiosis can exacerbate barrier dysfunction and inflammation. Collectively, these aging-induced deficits in digestion, barrier integrity, and microbiota pose a major challenge to intestinal and systemic health.

During the late laying period, Plant extracts offer a promising intervention strategy. For instance, they can enhance digestive enzyme activity, as shown with sorghum proanthocyanidins (Nyamambi et al., 2000) and oregano thymol (Feng et al., 2021b). Quercetin, for instance, has been shown to upregulate tight junction protein expression (Amevor et al., 2022). And plant extracts could also modulate gut microbiota composition and metabolism, demonstrated with glycerol monolaurate (Feng et al., 2021a) and various essential oils (Chen et al., 2020), particularly during the late laying period.

Among bioactive compounds, the natural diterpenoid sclareol presents unique comparative advantages due to its broad-spectrum biological activities (Caniard et al., 2012). First, it exhibits potent antimicrobial effects. Sclareol inhibits pathogens such as *E. gallinarum* (Bisio et al., 2020) and *P. anaerobius* (Souza et al., 2011) via diverse mechanisms including adenylate cyclase antagonism(SUBBIAH, 1999) and hemolysin inhibition(Ping et al., 2017), and its derivatives show enhanced antifungal activity(Ma et al., 2018). Second, sclareol alleviates inflammation in multiple organs. It modulates the MAPK/NF-κB pathway, inhibits inflammatory factors and angiogenesis, thereby reducing inflammatory damage and fibrosis in parenchymal organs such as the heart (Yang et al., 2023), liver (Ge et al., 2023), kidneys (Han et al., 2022), and Colon(Wang et al., 2024). Through anti-inflammatory pathways and inducing vascular pre-relaxation, it also mitigates edema in the lungs (Hsieh et al., 2017), feet (Huang et al., 2012), and joints (Tsai et al., 2018). Third, sclareol exerts in vivo antioxidant functions. Its mechanism may involve enhancing the activity of the endogenous antioxidant enzyme system (Wong et al., 2020). Similar to terpinen-4-ol, it may alleviate oxidative stress by chelating metal ions (Deen et al., 2023) and inhibit reactive oxygen species (ROS)-generating enzymes (Ravera et al., 2020).

Unlike many single-function extracts, sclareol’s broad-spectrum activity allows it to concurrently target intestinal injury, oxidative stress, and chronic inflammation, which is the key drivers of aging gut decline. Due to its lipophilic nature, it can diffuse directly through the lipid bilayer of the cell membrane. Given its bioactivity and absorbability, sclareol may provide an integrated solution for aging poultry. However, its application in poultry remains unexplored. This study aimed to evaluate sclareol's effects on production performance, egg SCFA content, and intestinal function in broiler breeders. We hypothesized that sclareol enhances intestinal function, helping animals cope with age-related challenges.

## Materials and methods

All procedures used in this experiment were approved by the Animal Welfare Committee of Sichuan Agricultural University (Approval No. 20240659).

### Experimental design, treatments and diets

At 42 weeks old, a total of 600 parent-generation Tianfu broiler breeders were weighed at the start of the experiment and randomly assigned to one of the five dietary treatments, ensuring that the average initial body weight and weight distribution were not significantly different among groups. Each treatment consisting of eight replicates with 15 birds per replicate. These groups included a control group (CON), a low-dose sclareol group (LSCL), a medium-dose sclareol group (MSCL), a high-dose sclareol group (HSCL), and a positive control group (ORE). The CON group was fed a basal diet, while the ORE group received the basal diet supplemented with 250 mg/kg of oregano oil (Thymol ≥9.6 %, Carvacrol ≥0.4 %, Moisture ≤10 %, CAS: 8007-11-2; Weifang Jiayijia Biotechnology Co., Ltd., China). The LSCL, MSCL, and HSCL groups were supplemented with 200, 400, and 800 mg/kg of sclareol (Purity >95 %, CAS: 515-03-7; Sichuan Huasheng Jiangyuan Fine Chemical Co., Ltd., China), respectively.

The dietary supplementation for each group was prepared daily. Sclareol (2,640, 5,280, or 10,560 mg, adjusted for purity) was dissolved in 30 mL of 95 % ethanol at 50°C, and the solution was stored in a dedicated spray bottle. This solution was evenly sprayed onto 100 g of basal diet and mixed to form a premix. The premix was then incorporated into the final diet (approx. 13.2 kg) by sequentially mixing with equal amounts of feed, followed by thorough mixing in a drum mixer (D30-B, Henan, Zhengzhou, Lima Machinery Co.,Ltd.) to ensure homogeneity. The final diet was spread flat for 3 hours to evaporate the ethanol, then stored in a cool, ventilated area. Prior to feeding, the diet was remixed and lightly misted with water to enhance powder adhesion and prevent segregation.

The diets were prepared in accordance with the nutritional guidelines of the NRC (1994) and the Chinese Feeding Standard for Chickens (NY/T 33-2004). Details of the basal diet's composition and nutritional content are shown in Table 1. The experiment included a 7-day acclimatization period and lasted for a total of 84 days. Conducted at Chengdu Chongzhou Xiangyu Agriculture and Animal Husbandry Co., Ltd., the study utilized a gradient single-cage rearing system. Throughout the trial, birds had unrestricted access to water, and feed was provided at set times, with strict compliance with sanitation and disinfection protocols.

**Table 1:** Ingredient composition of basal diet.

Ingredient	Content (%)
Corn (kg)	58.3
Soybean meal (kg)	28.3
Limestone (kg)	6.10
Soybean oil (kg)	3.00
Wheat bran (kg)	2.00
Dicalcium phosphate (kg)	0.60
Vitamin premix^1^ (kg)	
DL-Methionine (kg)	0.20
L-Lysine (kg)	0.20
Salt (kg)	0.30
Premix^2^ (kg)	1.00
Calculated composition	
Metabolizable Energy (MJ/kg)	12.57
Crude Protein (%)	16.63
Calcium (%)	3.50
Total Phosphorus (%)	0.62
Lysine (%)	0.83
Methionine + Cystine (%)	0.55

1 Complex vitamins are provided per kilogram of feed: VA 13500 IU, VD3 3600 IU, VE 36 IU, VK3 5 mg, VB1 5.0 mg, d-pantothenic acid 22 mg, nicotinamide 41.94 mg, folic acid 3 mg and biotin 0.25 mg.

2 The premix provides per kilogram of feed: Iron (Fe) as ferrous sulfate monohydrate (FeSO_4_·H_2_O): 100 mg; Copper (Cu) as copper(II) sulfate pentahydrate (CuSO_4_·5H_2_O): 8 mg; Manganese (Mn) as manganese sulfate monohydrate (MnSO_4_·H_2_O): 100 mg; Iodine (I) as potassium iodide (KI): 0.7 mg; Selenium (Se) as sodium selenite (Na_2_SeO_3_): 0.35 mg.

### Production performance

During the experimental period, the number of eggs laid, egg weight, and the number of deformed and cracked eggs were recorded daily at 9:00 AM for each replicate. Based on the collected data, the following parameters were calculated weekly for each group: feed-to-egg ratio, egg production rate, defective egg rate (including cracked, thin-shelled, and deformed eggs), and average qualified egg weight. The formulas used for calculations were as follows.

### Serum antioxidant indicators

On the 42nd and 84th days of the study, a single bird from each replicate group was chosen, and blood samples ranging from 3 to 5 mL were drawn from the wing vein. These samples were left at room temperature for half an hour before being centrifuged at 4°C and 3500 rpm for 15 minutes using a refrigerated centrifuge. The serum obtained was then stored at −80°C for later analysis. The serum concentrations of T-SOD, T-AOC, GSH, and MDA were determined by strictly adhering to the instructions provided with the assay kits from Nanjing Jiancheng Bioengineering Institute, located in Nanjing, Jiangsu Province, China.

### Egg quality, amino acid/fatty acid profile

At weeks 6 and 12, a random selection of 12 eggs from each treatment group was performed to evaluate egg quality. Egg quality parameters were assessed as follows. Shell strength (N) was determined using an eggshell strength tester, and shell thickness (mm) was measured with a micrometer. Egg weight (g) and shell weight were recorded using an electronic balance, and the shell ratio (%) was calculated as (shell weight / egg weight) × 100. The yolk index and egg shape index were derived as yolk height / yolk width and egg length / egg width, respectively, with all linear measurements taken using a vernier caliper. Yolk color was scored against the DSM Egg Color Fan. Average albumen height (mm) was determined at three equidistant points around the yolk using an albumen height gauge. The Haugh unit was calculated using the formula: 100 × log (H - 1.7 × *M*^0.37 + 7.6), where H is the average albumen height (mm) and M is the egg weight (g).

After 12 weeks, three eggs were randomly chosen from each treatment group. These eggs were cracked open, and the egg whites and yolks were thoroughly mixed to create whole egg liquid for analysis. The amino acids (AA) were analyzed according to GB 5009.124-2016, using an Essentia LC-16AAA amino acid analyzer (Shimadzu Corporation, Kyoto, Japan). The fatty acids (FA) were analyzed according to Method 2 of GB 5009.168-2016, using a 7890B gas chromatograph (Agilent Technologies, Inc., Santa Clara, CA, USA).

### Gut morphology analysis

On the 84th day of the experiment, three birds were randomly selected from each replicate and euthanized by exsanguination through the jugular vein. Laparotomy was performed to harvest the duodenum, ileum, and jejunum, and any adherent connective tissue and adipose were meticulously dissected away. Subject the specimens to fixation in 4 % formaldehyde solution for 24 hours, with one change of fixative. Following a seven-day fixation period, retrieve the tissues and excise approximately 1 cm sections from the relatively straight middle portions of each intestinal segment for subsequent paraffin embedding and microtomy. Tissue samples were imaged using a microscope, and intestinal morphology was examined using an Olympus upright microscope. OlyVIA 4.1.1 software was used to measure the height of 10 intact villi and the depth of their corresponding crypts in the intestinal tissue. The villus-to-crypt ratios and their average values were calculated.

### Gut mucosal antioxidant function

On days 42 and 84 of the experiment, one bird was selected from each replicate, with a total of eight birds per group. The birds were weighed and euthanized via jugular vein exsanguination, and the abdominal cavity was quickly opened to remove the duodenum, jejunum, and ileum intact. After removing the connective tissue and fat, the mucosal layers of the duodenum, jejunum, and ileum were weighed accurately. A 10 % intestinal tissue homogenate was prepared by adding nine volumes of 0.9 % saline solution to the tissue (weight (g): volume (mL) = 1:9) and homogenizing using a high-throughput tissue grinder at −20°C and 60 Hz for 100 s. The homogenate (400 μL) was centrifuged at 4°C and 2500–3000 rpm for 10 min, and the supernatant was collected for further analysis. The activities of T-SOD and T-AOC, as well as the levels of GSH and MDA in the duodenal, jejunal, and ileal tissues, were measured using assay kits (Nanjing Jiancheng Bioengineering Institute, Nanjing, Jiangsu Province, China) according to the manufacturer's instructions.

### Gut mucosal digestive enzyme content

On days 42 and 84 of the experiment, one bird was selected from each replicate, totaling eight birds per group. The birds were weighed, euthanized via jugular vein exsanguination, and the abdominal cavity was quickly opened to remove the duodenum, jejunum, and ileum intact. After removing the connective tissue and fat, the intestines were cut open and flattened, and the mucosal layer was scraped using a glass slide. Mucosal samples were separately harvested from the duodenum, jejunum and ileum. After longitudinal incision and gentle PBS rinsing, the mucus-epithelial layer was scraped with a glass slide, weighed, and diluted 1:9 (w/v) in ice-cold 0.9 % NaCl. Tissue was homogenized at –20 °C, 60 Hz for 100 s, then centrifuged at 4 °C, 2 500–3 000 × *g* for 10 min. The supernatant was collected for analysis. The activities of trypsin (TRY), α-amylase (AMS), and lipase (LPS) in the serum of 12-week-old broiler breeders were measured using assay kits (Nanjing Jiancheng Bioengineering Institute, Nanjing, Jiangsu Province, China), according to the manufacturer's instructions.

### Q-PCR determination of SREBF1, FATP4, and ACC expression in gut tissue

Using TRIzol reagent, total RNA was isolated from gut tissues, and its concentration was modified to a suitable amount with DEPC-treated water. The process of reverse transcription utilized a Real-Time PCR amplification system, adhering rigidly to the guidelines included in the reverse transcription kit. The quantitative real-time PCR (qPCR) process utilized a real-time fluorescence quantitative PCR device, employing SYBR Green PCR Mix as the fluorescent dye. The composition of the reaction system included a 10 μL blend (comprising 5 μL SYBR Green qPCR Master Mix, 1 μL of cDNA template, 2 μL each of forward and reverse primers, and 3 μL RNase-free water). The β-actin gene served as the internal benchmark, while the comparative expression rates of the target genes were determined through the ∆∆Ct technique. Table 2 enumerates the sequences of primers used for the specified genes.

**Table 2:** Genes and their primer sequence.

Gene^1^	Gene Bank ID	Primer sequence (5′−3′)	Product size (bp)
SREBF-1	NM_204126.3	F GCAGAAGAGCAAGTCCCTCAA R GGAGCCTACATCCGAGGG	130
FATP4	XM_046929199.1	F ATACCTCTGGCACTACGGGAAT R CATACATCACATCATCGGGTCT	117
ACC	NM_205505.2	F TTGTGGCACAGAAGAGGGAA R GTTGGCACATGGAATGGCAG	161
β-actin	NM_205518.2	F AACACCCACACCCCTGTGAT R TGAGTCAAGCGCCAAAAGAA	100

1 SREBF-1,sterol regulatory element binding transcription factor 1.FATP4,fatty acid transport protein 4.ACC,acyl-CoA carboxylase.

### Cecal content 16S rRNA sequencing

On the 84th day of the study, a single chicken from every duplicate was chosen at random and humanely euthanized through the exsanguination of the jugular vein. Immediately after, the abdominal cavity was unsealed, and the contents of the cecum were gathered. Using the SDS technique, genomic DNA was isolated from the specimens, which were evaluated for purity and concentration through agarose gel electrophoresis. A suitable quantity of DNA sample was portioned into a centrifuge tube and thinned down to 1 μg/μL with sterile water. For PCR amplification aimed at the V4 region of the 16S rRNA gene, diluted genomic DNA served as the template, utilizing barcoded specific primers and a High-Fidelity PCR Master Mix with GC Buffer to guarantee both efficiency and precision in amplification. Utilizing the TruSeq® DNA PCR-Free Sample Preparation Kit, a sequencing library was developed. Evaluation of the assembled library's quality was conducted through Qubit and qPCR quantification techniques. Following the verification of the library's quality, the sequencing process was executed using the NovaSeq 6000 system.

### Statistical analysis

Raw data underwent recording and initial computation using Microsoft Excel. The statistical evaluation was conducted utilizing the SPSS 18.0 software. Average values across groups were analyzed through one-way ANOVA, perform post-hoc analysis using Duncan's test, with outcomes presented as "mean ± standard error." A P-value less than 0.05 was employed to denote differences of statistical significance.

## Results

### Effects of dietary sclareol supplementation on the performance of broiler breeders in the late egg-laying period

**Fig. 1:**
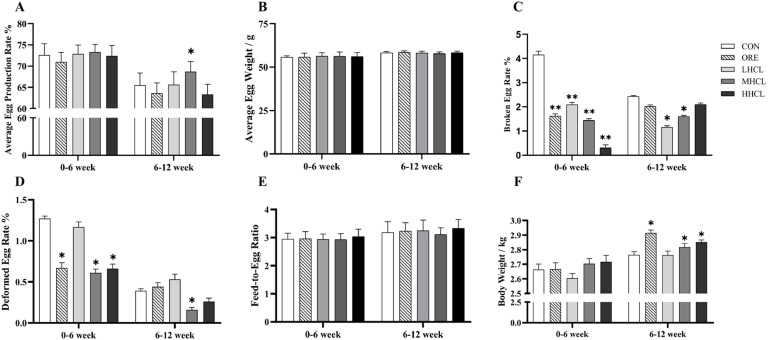
The Impact of Sclareol on the Productive Performance of Tianfu Broiler Breeders.

**Note:** A. Average Egg Production Rate (AEPR, %); B. Average Egg Weight (AEW, g); C. Broken Egg Rate (BER, %); D. Deformed Egg Rate (DER, %); E. Feed to Egg Ratio (FER, %); F. Body Weight (BW, kg).* indicates a significant difference between groups compared to the CON group (*P <* 0.05),** indicates an extremely significant difference between groups compared to the CON group (*P <* 0.01).

**Fig. 2:**
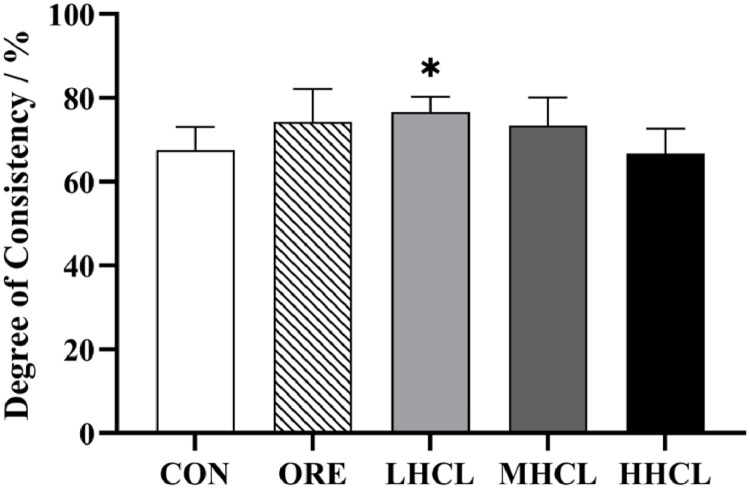
The Impact of Sclareol on the Uniformity of Tianfu Broiler Breeders.

**Note:** Uniformity (DC, %). * Indicates a significant difference between groups compared to the CON group (*P <* 0.05)

During the 0–6-week trial, the production indicators for each dose group showed significant changes (Fig. 1). Compared to the CON group, the average egg production rate increased significantly (*P* < 0.05), while the egg breakage rate decreased significantly (*P* < 0.01). The malformed egg rate in the MSCL and HSCL groups showed a significant increase (*P* < 0.05). No significant changes were observed in the average egg production rate, average egg weight, feed-to-egg ratio, and body weight. During the 6–12-week trial, compared to the CON group, the MSCL group showed a significant increase in average egg production rate (*P* < 0.05), while the LSCL and MSCL groups exhibited a significant decrease in egg breakage rate (*P* < 0.05). The malformed egg rate in the MSCL group decreased significantly (*P* < 0.05), and the body weight of the ORE, MSCL, and HSCL groups increased significantly (*P* < 0.05). No significant difference was observed about the feed-to-egg ratio in the MSCL and HSCL groups (*P* > 0.05). In addition, compared to the CON group, the uniformity of the flock in the LSCL group was significantly higher at 84 days (*P* < 0.05) (Fig. 2).

### Effects of dietary sclareol supplementation on serum antioxidant capacity and egg quality of broiler breeders in the late laying period

**Fig. 3:**
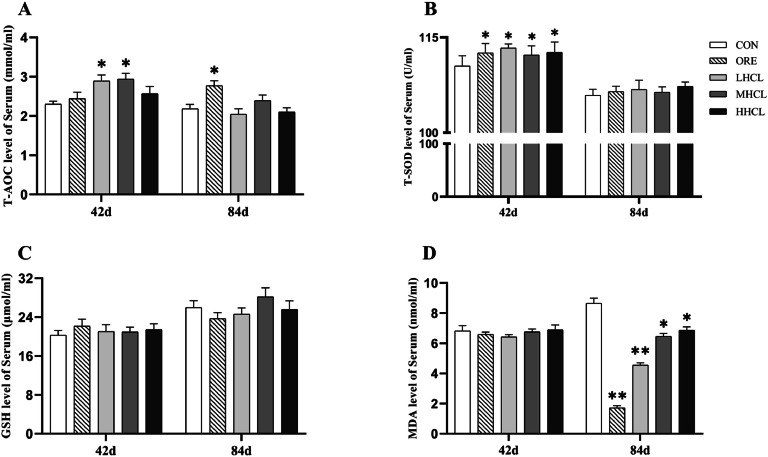
The Impact of Sclareol on the Antioxidant Function of Serum in Tianfu Broiler Breeders.

**Note:** A. Total Antioxidant Capacity (T-AOC, mmol/mL), B. Total Superoxide Dismutase (T-SOD, U/mL), C. Glutathione (GSH, μmol/g), D. Malondialdehyde (MDA, μmol/g). * Indicates a significant difference between groups compared to the CON group (*P <* 0.05), ** indicates an extremely significant difference between groups compared to the CON group (*P <* 0.01).

Dietary supplementation of oregano thymol was shown to improve serum antioxidant function in Tianfu broiler breeders (Fig. 3). Compared with the CON group, the serum T-AOC level of the LSCL group and the MSCL group was significantly increased (*P* < 0.05) on the 42nd day of the study, and the serum T-SOD activity of each experimental group was highly significant (*P* < 0.05). The remaining indices did not change significantly (*P* > 0.05). At the 84th day of the study, the serum T-AOC level of the ORE group was significantly higher, the serum MDA level in the MSCL and LSCL groups was significantly lower (*P* < 0.05), serum MDA level in ORE and LSCL groups was highly significant (*P* < 0.01).

The quality of the eggs is displayed in Table 3. At day 42 of the experiment, all experimental groups of sclareol had no significant effect on all egg quality indexes of broiler breeders in the middle stage of laying (*P* > 0.05). However, at the 84th day of the experiment, the eggshell thickness of breeder eggs in the LSCL group and MSCL group exhibited a significant enhancement (*P* < 0.05). The eggshell thickness in the HSCL group did not change significantly, but it demonstrated a significant improvement in the HHU value (*P* < 0.05). The findings suggest that sclareol can enhance eggshell quality and freshness.

**Table 3:** The Impact of Sclareol on Egg Quality in Tianfu Broiler Breeders.

Organ Index	CON	ORE	DHCL	MHCL	HHCL	P-value
42d						
Egg weight/g	57.25 ± 1.81	57.19 ± 2.41	58.10 ± 3.68	58.20 ± 2.37	59.25 ± 1.80	0.326
Eggshell strength/N	29.58 ± 8.19	31.58 ± 8.26	29.98 ± 5.59	31.18 ± 5.58	33.35 ± 5.96	0.749
Eggshell thickness/mm	0.32 ± 0.03	0.34 ± 0.02	0.31 ± 0.03	0.32 ± 0.02	0.34 ± 0.02	0.060
Yolk color	6.92 ± 0.79	7.42 ± 0.79	7.67 ± 1.15	7.58 ± 0.79	7.80 ± 0.92	0.171
Egg shape index	1.32 ± 0.03	1.35 ± 0.14	1.31 ± 0.04	1.31 ± 0.04	1.31 ± 0.03	0.646
Shell ratio/%	0.12 ± 0.01	0.12 ± 0.01	0.12 ± 0.01	0.12 ± 0.01	0.12 ± 0.01	0.797
Yolk index	0.32 ± 0.03	0.32 ± 0.04	0.33 ± 0.03	0.34 ± 0.04	0.35 ± 0.02	0.188
Haugh unit	85.10 ± 2.22	84.90 ± 2.86	82.46 ± 2.46	84.96 ± 2.54	84.79 ± 2.79	0.159
84d						
Egg weight/g	58.01 ± 1.83	57.35 ± 1.56	57.37 ± 2.82	59.08 ± 2.51	60.92 ± 2.59	0.270
Eggshell strength/N	30.34 ± 6.31	34.28 ± 9.38	34.55 ± 6.92	36.26 ± 8.40	33.63 ± 6.89	0.443
Eggshell thickness/mm	0.32 ± 0.02^b^	0.33 ± 0.03^b^	0.37 ± 0.03^a^	0.37 ± 0.04^a^	0.34 ± 0.02^b^	0.006
Yolk color	7.50 ± 0.85	7.22 ± 0.44	7.33 ± 0.50	7.60 ± 0.70	7.50 ± 0.80	0.769
Egg shape index	1.36 ± 0.05	1.36 ± 0.04	1.34 ± 0.04	1.34 ± 0.06	1.33 ± 0.05	0.519
Shell ratio/%	0.12 ± 0.01	0.12 ± 0.01	0.12 ± 0.00	0.11 ± 0.01	0.12 ± 0.01	0.050
Yolk index	0.35 ± 0.04	0.33 ± 0.03	0.35 ± 0.01	0.35 ± 0.03	0.33 ± 0.03	0.187
Haugh unit	83.31 ± 2.84^b^	86.48 ± 3.18^ab^	87.68 ± 4.83^a^	88.46 ± 4.66^a^	84.60 ± 3.21^b^	0.048

a,b,c: different letter marks represent significant differences (*P* < 0.05).

The results were means ± standard deviation, with 6 replicates per group (*n* = 6).

As demonstrated in Table 4, all experimental groups of sclareol exhibited significant effects on multiple amino acid and fatty acid indices in whole egg solution at day 84 of the experiment, in comparison to the CON group. A significant elevation in essential amino acids was observed in the MSCL group compared to the CON group, with substantial increases in threonine (0.598 ± 0.009 vs 0.548 ± 0.009, *P* < 0.05) and valine (0.842 ± 0.013 vs 0.768 ± 0.013, *P* < 0.05). The content of lysine increased significantly (0.973 ± 0.014 vs. 0.867 ± 0.015, *P* < 0.001), as did the content of valine (0.842 ± 0.013 vs. 0.768 ± 0.013, *P* < 0.05). A significant increase in threonine content was observed in the MSCL group (0.598 ± 0.009 vs. 0.548 ± 0.009, *P* < 0.05), and a significant increase in lysine content was also observed in the MSCL group (0.973 ± 0.014 vs. 0.867 ± 0.015, *P* < 0.001) compared to the CON group. Levels of glutamic acid (1.633 ± 0.024 vs. 1.485 ± 0.025, *P* < 0.01) and arginine (0.862 ± 0.013 vs. 0.758 ± 0.013, *P* < 0.001) were observed. These findings indicate that sclareol may influence the deposition of essential amino acids in the egg by modulating protein metabolism. Non-essential amino acids exhibited differential regulation, with proline demonstrating a significant reduction in the HSCL group (0.365 ± 0.005 vs 0.419 ± 0.007, *P* < 0.001). Furthermore, methionine levels were reduced in the LSCL group (0.392 ± 0.016 vs 0.419 ± 0.007, *P* < 0.05), suggesting that high doses of sclareol may inhibit specific amino acid synthesis pathways. Total amino acids exhibited a significant increase in the MSCL group (12.391 ± 0.199 vs 11.357 ± 0.195, *P <* 0.01), representing a 9.1 % increase compared to the CON group and a substantial enhancement over other treatment groups (*P* < 0.05). Concurrently, there was a decline in saturated fatty acid (SFA) components. Among these, tetra decanoic acid (C14:0) demonstrated a significant decrease in the HSCL group (0.023 ± 0.002 vs 0.028 ± 0.001, *P* < 0.001), and hexadecenoic acid (C16:1) was decreased by 28.7 % in the LSCL group (0.170 ± 0.002 vs 0.188 ± 0.005, *P* < 0.001).

**Table 4:** Effects of Sclareol on the Amino Acid and Fatty Acid Content of Eggs from Tianfu Broiler Breeders.

Acids	CON	ORE	DHCL	MHCL	HHCL	P-value
Aspartic acid	1.196 ± 0.020ᵇ	1.298 ± 0.019ᵃ	1.206 ± 0.050ᵇ	1.237 ± 0.018ᵇ	1.257 ± 0.019ᵃᵇ	0.009
Threonine	0.548 ± 0.009ᵇ	0.598 ± 0.009ᵃ	0.567 ± 0.024ᵇ	0.558 ± 0.008ᵇ	0.588 ± 0.009ᵃ	0.005
Serine	0.718 ± 0.012ᵇ	0.740 ± 0.011ᵇ	0.742 ± 0.031ᵇ	0.619 ± 0.009ᶜ	0.842 ± 0.013ᵃ	0.001
Glutamic acid	1.485 ± 0.025ᵇ	1.633 ± 0.024ᵃ	1.525 ± 0.064ᵇ	1.491 ± 0.022ᵇ	1.562 ± 0.023ᵃᵇ	0.003
Glycine	0.399 ± 0.007ᶜ	0.426 ± 0.006ᵃ	0.392 ± 0.016ᶜ	0.395 ± 0.006ᶜ	0.406 ± 0.006ᵇ	0.008
Alanine	0.698 ± 0.012ᶜ	0.761 ± 0.011ᵃ	0.701 ± 0.029ᶜ	0.740 ± 0.011ᵇ	0.690 ± 0.010ᶜ	0.001
Valine	0.768 ± 0.013ᶜ	0.842 ± 0.013ᵃ	0.773 ± 0.032ᶜ	0.801 ± 0.012ᵇ	0.771 ± 0.011ᶜ	0.002
Methionine	0.419 ± 0.007ᵇ	0.436 ± 0.006ᵃ	0.392 ± 0.016ᶜ	0.416 ± 0.006ᵇ	0.416 ± 0.006ᵇ	0.003
Isoleucine	0.618 ± 0.011ᶜ	0.679 ± 0.010ᵃ	0.649 ± 0.027ᵇ	0.659 ± 0.010ᵇ	0.608 ± 0.009ᶜ	0.001
Leucine	1.027 ± 0.018ᶜ	1.146 ± 0.017ᵃ	1.093 ± 0.046ᵇ	1.115 ± 0.017ᵇ	1.014 ± 0.015ᶜ	0.001
Tyrosine	0.488 ± 0.008ᶜ	0.548 ± 0.008ᵃ	0.536 ± 0.022ᵇ	0.527 ± 0.008ᵇ	0.477 ± 0.007ᶜ	0.001
Phenylalanine	0.658 ± 0.011ᵇ	0.700 ± 0.010ᵃ	0.639 ± 0.027ᶜ	0.669 ± 0.010ᵇ	0.608 ± 0.009ᶜ	0.001
Lysine	0.867 ± 0.015ᶜ	0.973 ± 0.014ᵃ	0.938 ± 0.039ᵇ	0.933 ± 0.014ᵇ	0.862 ± 0.013ᶜ	0.001
Histidine	0.289 ± 0.005ᵇ	0.314 ± 0.005ᵃ	0.299 ± 0.013ᵇ	0.294 ± 0.004ᵇ	0.294 ± 0.004ᵇ	0.011
Arginine	0.758 ± 0.013ᶜ	0.862 ± 0.013ᵃ	0.845 ± 0.035ᵇ	0.831 ± 0.012ᵇ	0.771 ± 0.011ᶜ	0.001
Proline	0.419 ± 0.007ᵇ	0.446 ± 0.007ᵃ	0.433 ± 0.018ᵇ	0.446 ± 0.007ᵃ	0.365 ± 0.005ᶜ	0.001
Total amino acids	11.357 ± 0.195ᵇ	12.391 ± 0.199ᵃ	11.736 ± 0.484ᵇ	11.742 ± 0.161ᵇ	11.539 ± 0.158ᵇ	0.008
C14:0	0.028 ± 0.001ᵇ	0.031 ± 0.000ᵃ	0.025 ± 0.000ᶜ	0.024 ± 0.000ᶜ	0.023 ± 0.002ᶜ	0.001
C14:1	0.005 ± 0.000ᵇ	0.006 ± 0.000ᵃ	0.004 ± 0.000ᶜ	0.005 ± 0.000ᵇ	0.006 ± 0.001ᵃ	0.001
C15:0	1.907 ± 0.046ᵇ	2.013 ± 0.022ᵃ	1.891 ± 0.021ᵇ	1.891 ± 0.021ᵇ	2.079 ± 0.192ᵃ	0.092
C16:0	0.798 ± 0.019ᶜ	0.909 ± 0.010ᵇ	0.781 ± 0.009ᶜ	0.900 ± 0.010ᵇ	0.960 ± 0.089ᵃ	0.001
C16:1	0.025 ± 0.001ᶜ	0.043 ± 0.000ᵃ	0.024 ± 0.000ᶜ	0.028 ± 0.000ᵇ	0.049 ± 0.005ᵃ	0.001
C17:0	6.762 ± 0.164ᵇ	6.899 ± 0.076ᵃ	6.689 ± 0.073ᵇ	6.735 ± 0.074ᵇ	6.916 ± 0.637ᵃ	0.842
C18:0	1.196 ± 0.020ᵇ	1.298 ± 0.019ᵃ	1.206 ± 0.050ᵇ	1.237 ± 0.018ᵇ	1.257 ± 0.019ᵃᵇ	0.009
C18:1n9t	0.548 ± 0.009ᵇ	0.598 ± 0.009ᵃ	0.567 ± 0.024ᵇ	0.558 ± 0.008ᵇ	0.588 ± 0.009ᵃ	0.005
C18:1n9c	0.718 ± 0.012ᵇ	0.740 ± 0.011ᵇ	0.742 ± 0.031ᵇ	0.619 ± 0.009ᶜ	0.842 ± 0.013ᵃ	0.001
C18:2n6c	1.485 ± 0.025ᵇ	1.633 ± 0.024ᵃ	1.525 ± 0.064ᵇ	1.491 ± 0.022ᵇ	1.562 ± 0.023ᵃᵇ	0.003
C18:3n3	0.399 ± 0.007ᶜ	0.426 ± 0.006ᵃ	0.392 ± 0.016ᶜ	0.395 ± 0.006ᶜ	0.406 ± 0.006ᵇ	0.008
C18:3n6	0.698 ± 0.012ᶜ	0.761 ± 0.011ᵃ	0.701 ± 0.029ᶜ	0.740 ± 0.011ᵇ	0.690 ± 0.010ᶜ	0.001
C20:1	0.768 ± 0.013ᶜ	0.842 ± 0.013ᵃ	0.773 ± 0.032ᶜ	0.801 ± 0.012ᵇ	0.771 ± 0.011ᶜ	0.002
C20:2	0.419 ± 0.007ᵇ	0.436 ± 0.006ᵃ	0.392 ± 0.016ᶜ	0.416 ± 0.006ᵇ	0.416 ± 0.006ᵇ	0.003
C20:3n6	0.618 ± 0.011ᶜ	0.679 ± 0.010ᵃ	0.649 ± 0.027ᵇ	0.659 ± 0.010ᵇ	0.608 ± 0.009ᶜ	0.001
C20:3n3	1.027 ± 0.018ᶜ	1.146 ± 0.017ᵃ	1.093 ± 0.046ᵇ	1.115 ± 0.017ᵇ	1.014 ± 0.015ᶜ	0.001
C22:6n3	0.488 ± 0.008ᶜ	0.548 ± 0.008ᵃ	0.536 ± 0.022ᵇ	0.527 ± 0.008ᵇ	0.477 ± 0.007ᶜ	0.001
Total fatty acids	0.658 ± 0.011ᵇ	0.700 ± 0.010ᵃ	0.639 ± 0.027ᶜ	0.669 ± 0.010ᵇ	0.608 ± 0.009ᶜ	0.001

a,b,c: different letter marks represent significant differences (*P* < 0.05).

The results were means ± standard deviation, with 6 replicates per group (*n* = 6).

**Fig. 4:**
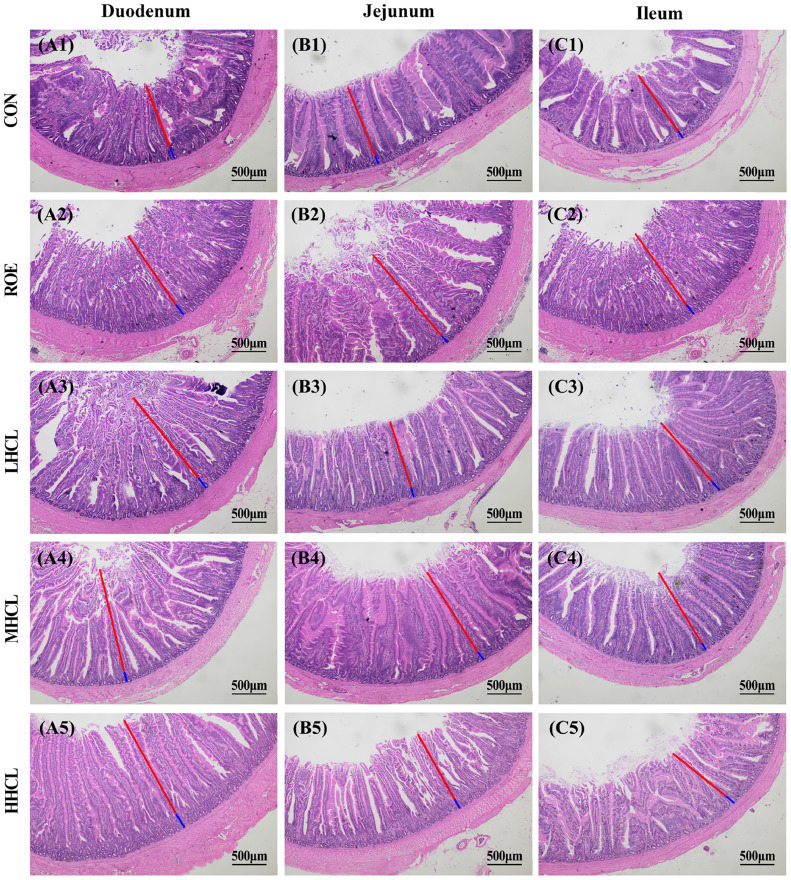
Intestinal Morphological Structure of Duodenum, Jejunum, and Ileum in Broiler Breeders of Different Groups.

**Note:** A1-A5 represent the duodenum of each group, B1-B5 represent the jejunum of each group, and C1-C5 represent the ileum of each group. The scale bar is 500 μm, where the blue label represents villus height, and the red label represents crypt depth.

**Fig. 5:**
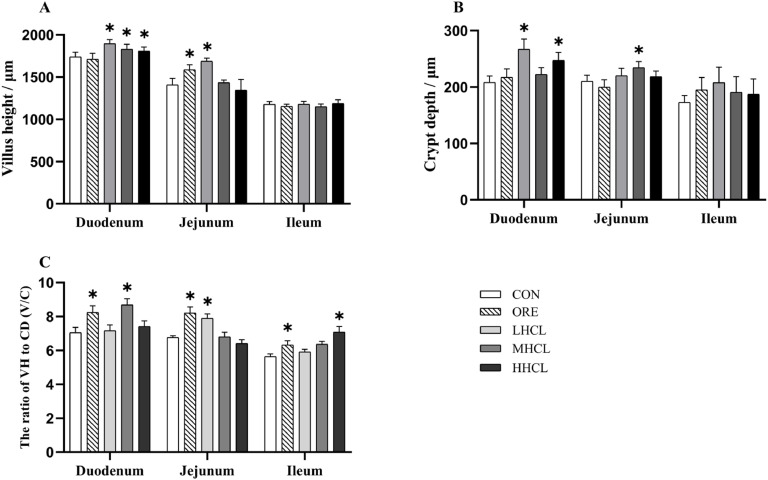
Villus Height, Crypt Depth, and Villus-to-Crypt Ratio in the Duodenum, Jejunum, and Ileum of Broiler Breeders Across Different Groups.

**Note:** A. Villus Height (VH, μm), B. Crypt Depth (CD, μm), C. Villus to Crypt Ratio (V/C). * Indicates a significant difference between groups compared to the CON group (*P <* 0.05).

Histological sections (H.E. 500 × magnification) of the intestines were collected at day 84 of the experiment, and quantitative statistics are shown in Fig. 4, Fig. 5. In the histological sections, different degrees of changes were observed in various segments of the intestine. The quantitative statistical results showed that compared with the CON group, and the height of jejunal villi was significantly higher (*P <* 0.05) in the ORE and HSCL groups. The depth of duodenal crypts was significantly higher (*P <* 0.05) in the ORE and the HSCL group, while the depth of jejunal crypts was significantly higher (*P <* 0.05) in the MSCL group. Compared to the CON group, the duodenal villous-to-crypt ratio was significantly higher In the ORE and MSCL groups, the jejunal villous-to-crypt ratio was significantly higher (*P <* 0.05), and in the ORE and HSCL groups, the ileal villous-to-crypt ratio was significantly higher (*P <* 0.05).

### Effects of dietary sclareol supplementation on intestinal function of broiler breeders in the late laying period

**Fig. 6:**
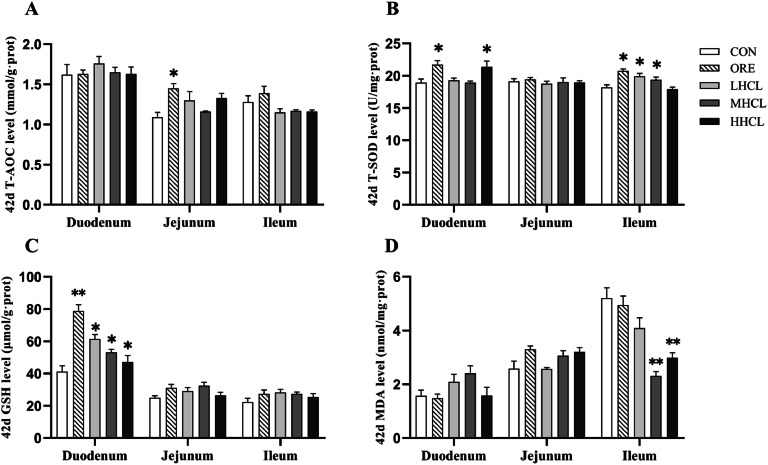
The Impact of Sclareol on Intestinal Antioxidant Function in Tianfu Broiler Breeders (42d).

**Note:** A. Total Antioxidant Capacity (T-AOC, mmol/mL), B. Total Superoxide Dismutase (T-SOD, U/mL), C. Glutathione (GSH, μmol/g), D. Malondialdehyde (MDA, μmol/g). * Indicates a significant difference between groups compared to the CON group (*P <* 0.05),** indicates an extremely significant difference between groups compared to the CON group (*P <* 0.01).

**Fig. 7:**
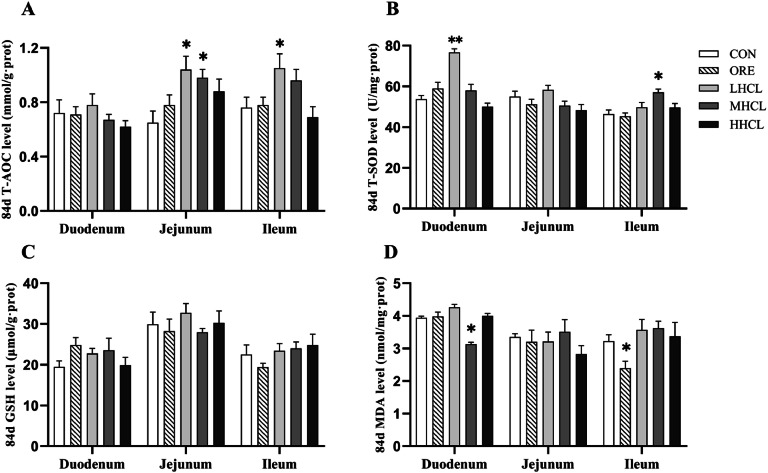
The Impact of Sclareol on Intestinal Antioxidant Function in Tianfu Broiler Breeders (84d).

**Note:** A. Amylase (AMS, U/mg.gprot), B. Trypsin (TRY, U/mg.gprot), C. Lipase (LPS, U/mg.gprot).* indicates a significant difference between groups compared to the CON group (*P <* 0.05).

The results of duodenal antioxidant indexes are shown in Fig. 6, Fig. 7. At the 42nd day of the trial, in comparison with the CON group, the duodenal T-SOD activity in the HSCL and ORE groups was found to be significantly higher (*P* < 0.05), the duodenal GSH level in the ORE group was found to be significantly higher (*P* < 0.01), and the duodenal GSH level in each dose group of the sclareol group was found to be significantly higher (*P* < 0.05). The jejunal T-AOC activity in the ORE group was significantly higher (*P* < 0.05); ileal T-SOD activity was significantly higher in the ORE, LSCL and MSCL groups (*P* < 0.05), and ileal MDA content was highly significantly lower in the MSCL and HSCL groups (*P* < 0.01). The remaining indexes demonstrated no substantial alterations (*P* > 0.05). At the 84th day of the test, compared with the CON group, the duodenal T-SOD activity of the LSCL group was found to be significantly higher (*P* < 0.01), and the duodenal MDA content of the MSCL group was significantly lower (*P* < 0.05). Furthermore, the jejunal T-AOC level of the MSCL group and the HSCL group was significantly higher (*P* < 0.05), and the jejunal MDA content of the ORE group was significantly lower (*P* < 0.05). The ileal T-AOC level was found to be significantly higher in the MSCL group (*P* < 0.05), and ileal T-SOD activity was found to be significantly higher in the MSCL group (*P* < 0.05). The remaining indexes did not demonstrate a statistically significant difference; however, the GSH content of all intestines in each experimental group exhibited a tendency to increase (*P* < 0.05). The results indicated that sclareol could increase the intestinal antioxidant capacity of Tianfu broiler breeders in the late laying stage.

**Fig. 8:**
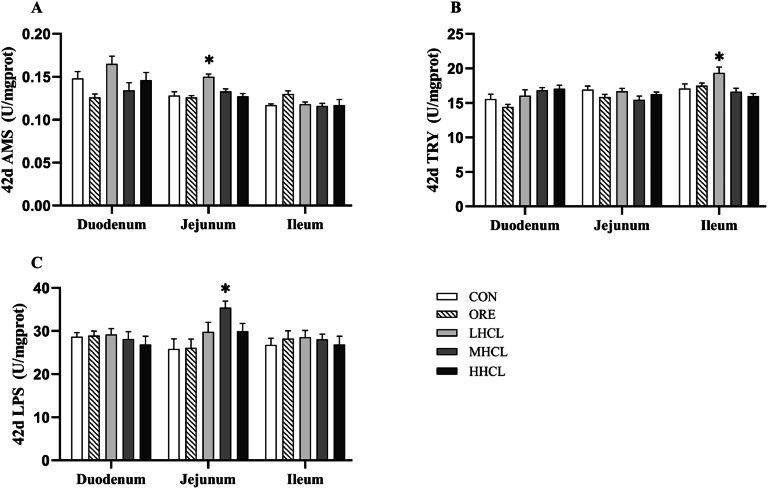
The Impact of Sclareol on Digestive Enzyme Content of Intestinal Mucosal in Tianfu Broiler Breeders (42d).

**Note:** A. Amylase (AMS, U/mg.gprot), B. Trypsin (TRY, U/mg.gprot), C. Lipase (LPS, U/mg.gprot). * Indicates a significant difference between groups compared to the CON group (*P <* 0.05).

**Fig. 9:**
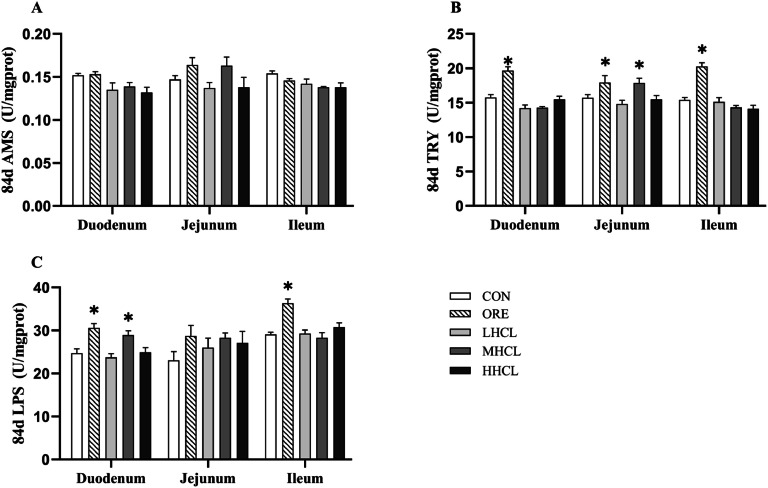
The Impact of Sclareol on Digestive Enzyme Content of Intestinal Mucosal in Tianfu Broiler Breeders (84d).

**Note:** A. Amylase (AMS, U/mg.gprot), B. Trypsin (TRY, U/mg.gprot), C. Lipase (LPS, U/mg.gprot).* indicates a significant difference between groups compared to the CON group (*P <* 0.05).

The mucosal digestive enzyme content of each intestinal segment is shown in Fig. 8, Fig. 9. Compared with the CON group, at 42 d, the jejunal mucosal α-amylase content was found to be significantly lower in the MSCL and HSCL groups (*P* < 0.001), and the jejunal mucosal lipase content was found to be significantly higher in the MSCL group (*P* < 0.05); at 84 d, no significant difference in the content of digestive enzymes of each intestinal segment among the test groups of sclareol was observed (*P* < 0.05).

**Fig. 10:**
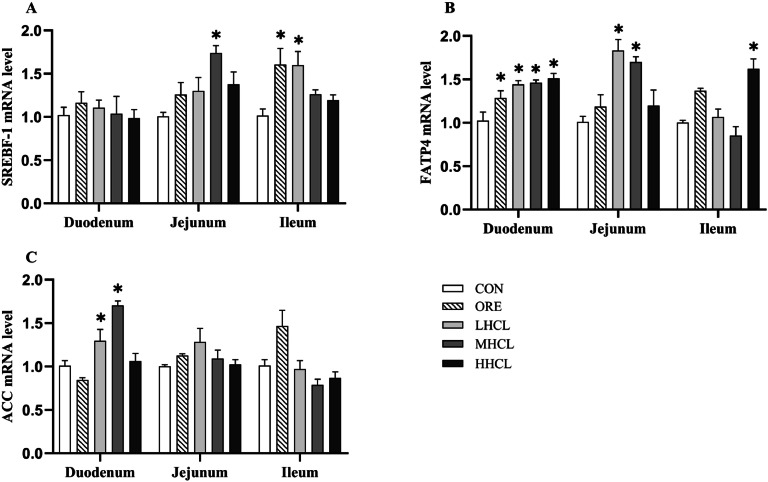
The mRNA levels of SREBF-1, FATP4, and ACC in the duodenum, jejunum, and ileum.

**Note:** A. Sterol Regulatory Element-Binding Protein 1 (SREBF-1), B. Fatty Acid Transport Protein 4 (FATP4), C. Acetyl-CoA Carboxylase Alpha (ACC). * Indicates a significant difference between groups compared to the CON group (*P <* 0.05).

The gene expression analysis revealed significant upregulation of key factors involved in fatty acid absorption and synthesis (Fig. 10). Specifically, duodenal FATP4 expression was significantly increased (*P* < 0.05) in all sclareol-supplemented groups compared to the CON group. In the jejunum, both FATP4 and SREBF-1 expression were significantly elevated (*P* < 0.05) in the MSCL group; notably, increased jejunal FATP4 was also observed in the LSCL group. In the ileum, SREBF-1 expression was significantly higher (*P* < 0.05) in the ORE and LSCL groups than in the CON group.

### Effects of dietary sclareol supplementation on the cecum microbiome of late-laying broiler breeders

**Fig. 11:**
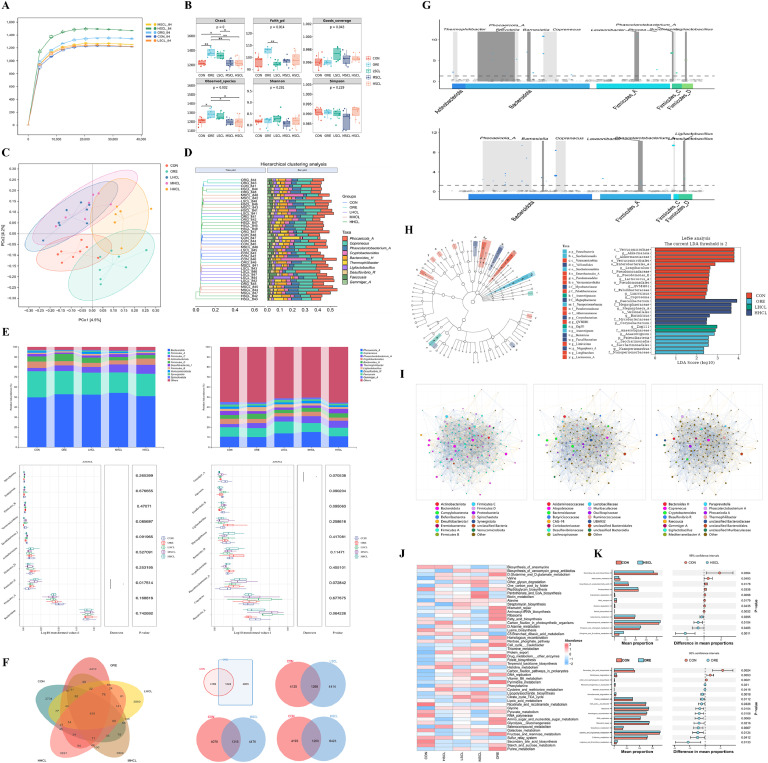
The Impact of Sclareol on Gut Microbiota of Tianfu Broiler Breeders.

**Note:** A. Sample dilution curve, B. Box plots of Alpha index, C. Beta diversity analysis based on Jaccard, D. Hierarchical clustering analysis. Relative abundance of species composition at the phylum and genus levels and Anova-based differential analysis, F. Veen map, G. MetagenomeSeq analysis, H. LDA effect size (LEfSe) analysis of differential microorganisms, I. Microbial interactions analysis, J. Metabolic Pathway Statistics, K. Functional Predictions.

Quality screening was conducted by sampling 40 cecal content specimens. A selection of sequences was randomly chosen, and the sequencing depth was indicated by the correlation between the number of sequence entries and the number of OUTs. The dilution curves for each group approached a plateau, suggesting that the sequencing depth had essentially captured all species present in the samples (Fig. 11A).

Alpha diversity analysis revealed that both the Chao1 and Observed species indices were significantly higher (*P* < 0.05), and the Goods coverage index was highly significant (*P* < 0.01) in the ORE group compared to the control group; the Chao1 index and Goods coverage index were also significantly higher (*P* < 0.05) in the LSCL group (Fig. 11B). The PCoA plot illustrated the outcomes of the Beta diversity analysis (Fig. 11C). It was observed that the samples from each group had minimal overlap, with the confidence intervals of the LSCL and MSCL groups being closer, indicating a shared cross-over position, which suggested a certain degree of similarity in the impact of low and medium doses of sclareol on the structure of cecal microbiota. In contrast, the ranges of all other groups were more dispersed with less overlap, indicating variability in β-diversity between the test groups and the CON group. Hierarchical cluster analysis showed that the LSCL and MSCL group samples tended to cluster together, while the ORE group was more distanced from the LSCL and MSCL groups compared to the CON group (Fig. 11D). This indicated that sclareol increased the Beta diversity of the microbiota, with the most pronounced effect observed in the LSCL group compared to the MSCL group.

Fig. 11E displays the compositional changes in the top 10 % of cecal microbial abundance levels. At the phylum level, the dominant phylum in the control group was Anabaena (49.72 %), followed by the Thick-walled phylum (35.84 %) and Actinobacteria (6.32 %), etc. In the ORE group, the dominant phylum was Anabaena (52.95 %), followed by the Thick-walled phylum (36.81 %) and Actinobacteria (4.56 %), etc. Compared to the CON group, the abundance of the Anabaena phylum increased in all dose groups, the abundance of the Thick-walled phylum showed no significant difference, and the ratio of Anabaena to Thick-walled phylum, F/B, increased, with the abundance of the Thick-walled phylum C taxa being significantly higher in the MSCL and HSCL groups (*P* < 0.05). At the genus level, the former abundances included *Phocaeicola, Coprenecus, Cryptobacteroides, Thermophilibacter*, etc., where the abundance of Gemmiger was significantly higher in the HSCL group compared to the CON group (*P* < 0.05), and the LSCL group showed a tendency towards increased *Phascolarctobacterium* and *Phocaeicola* abundance in the MSCL group (*P* > 0.05).

Among the five groups, a two-by-two comparison revealed that each group had more unique ASVs than the CON group (Fig. 11F). *MetagenomeSeq* differential abundance analysis indicated that, compared to the CON group, both the MSCL and HSCL groups exhibited the highest number of ASVs in *Phocaicola* as well as the highest relative abundance of ASVs in *Coprenecus*, and the concentration of differential ASVs in the MSCL and HSCL in *Thermophilibacter, Prevotella*, and *Succinivibrio* (Fig. 11G). Notably, the primary function of *Phocaicola* and *Prevotella* is to produce short-chain fatty acids (SCFAs).

Setting a linear discriminant analysis (LDA) value of 2, LEfSe analysis demonstrated that at the genus level, the differential groups in the CON group were *Akkermansia, Longibaculum, Pseudomonas-E, Lactococcus-A, QVMHO1, Limivicinus*, and *Coprosoma*; the ORE group included Anaerotignum, *Nanoperiomorbus*; the LSCL group had differential organisms *Zag111*; and the HSCL group had differential organisms *Faecalibacterium, Megasphaera A, Bariatricus, Coryebacterium* (Fig. 11H).

Consistency in the distribution of the groups belonging to the central node was observed in the colony interaction diagrams at the phylum, family, and genus levels (Fig. 11I). At the phylum level, it was predominantly short-chain fatty acid-producing Bacteroidota and Firmicutes A taxa that included genera such as Lactobacillus spp. and Bifidobacterium spp. At the family level, Bacteroidaceae and Oscillospiraceae were expressed, whose functions are the breakdown of indigestible polysaccharides to regulate host metabolism and the production of butyric acid, respectively. At the genus level, Coprenecus, Cryptobacteroides, Phascolarctobacterium, and Phocaeeicola were the main core genera.

The metabolic pathways of the cecum contents flora were predicted based on the ASV abundance of each species in the 16SrDNA sequencing results, and a heat map of metabolic pathway abundance was drawn based on the EC-normalized data from the PICRUSt2 analysis (Fig. 11J). The results indicated that the microbial community covered a wide range of functions from biosynthesis to degradation, energy metabolism to detoxification. Compared with the CON group, the oregano thymol group (ORE) was up-regulated in various amino acid metabolisms, vitamin metabolism, glucose metabolism, and other pathways such as tryptophan metabolism, phenylalanine metabolism, tyrosine metabolism, fatty acid metabolism, and energy metabolism, whereas it was down-regulated in ribosomal biosynthesis, rRNA biosynthesis, and protein synthesis. The sclareol dosage group was up-regulated in fatty acid metabolism and energy metabolism, whereas it was down-regulated in ribosome biosynthesis, protein synthesis, and other pathways were down-regulated.

Nonparametric STAMP analysis was conducted between the HSCL, ORE, and CON groups (Fig. 11K). The following eight pathways were significantly downregulated in the HSCL group compared to the CON group: secondary bile acid biosynthesis, β-lactam resistance, unsaturated fatty acid biosynthesis, photosynthesis, caprolactam degradation, RNA transport, atrazine degradation, and steroid biosynthesis. Additionally, the HSCL group significantly upregulated the following four pathways: β-alanine metabolism, selenium compound metabolism, inulin and chlorophyll metabolism, and d-arginine and d-ornithine metabolism. Compared with the CON group, the ORE group significantly downregulated the following three pathways: secondary bile acid biosynthesis, nitrotoluene degradation, and penicillin and cephalosporin biosynthesis. While the following thirteen pathways were significantly up-regulated: ribosome biosynthesis in eukaryotes, beet pigment biosynthesis, aminobenzoic acid degradation, purine metabolism, cell cycle-stalker bacillus, ribosomes, homologous recombination, DNA replication, mismatch repair, glutathione metabolism, d-glutamine and d-glutamate metabolism, d-alanine metabolism, and d-arginine and d-ornithine metabolism.

## Discussion

In the present study, long-term dietary supplementation with 400 mg/kg of sclareol during the late laying period improved the overall production performance of Tianfu broiler breeders. Specifically, it significantly increased the egg production rate and body weight while reducing the rates of broken and misshapen eggs, with the most pronounced effects observed in the later supplementation phase—likely due to enhanced eggshell thickness, which may also provide potential benefits in shielding embryos from external microorganisms. Concurrently, our study provides the first report on the modulatory effects of dietary sclareol on the gut microbiota in poultry. Sclareol treatment increased microbial diversity, enriched core short-chain fatty acid-producing genera, and significantly upregulated the expression of key intestinal fatty acid transport and metabolism genes (FATP4 and *SREBF-1*), which was associated with promoted deposition of beneficial fatty acids and amino acids in eggs. We hypothesize that these effects are mediated through sclareol’s influence on the gut microbiota and host lipid metabolism. Furthermore, sclareol enhanced intestinal digestive enzyme activity and improved villus architecture (villus height-to-crypt depth ratio), likely attributable to its ability to elevate intestinal antioxidant enzyme activity in aged breeders, thereby preserving intestinal morphology and optimizing the mucosal environment. Collectively, the improved production performance in late-phase broiler breeders appears to stem from the dual enhancement of gut microbial ecology and digestive function. Follow-up metabolomic studies are warranted to further elucidate the underlying pathways. This work confirms the intestinal efficacy of sclareol and provides pioneering evidence for the effectiveness of a diterpene alcohol in poultry production.

Egg production rate and body weight are the most critical parameters for evaluating the production performance of broiler breeders during the breeding process. Indicators such as average egg weight and cracked egg rate are closely related to the secretory functions of the ovaries and oviducts, reflecting the quality of ovulation in breeder hens to some extent (Nie et al., 2024) . Uniformity, which measures the variation in body weight within a flock, reflects the consistency of sexual maturity and growth development among individuals. The results indicated that dietary supplementation with 200 mg/kg sclareol significantly reduced the rates of cracked and deformed eggs and improved flock uniformity at 42 and 84 days. Meanwhile, 400 mg/kg sclareol significantly increased egg production rate and body weight while reducing cracked and deformed egg rates at 84 days, suggesting that 200–400 mg/kg is the optimal dosage range. Additionally, with the addition of sclareol to the feed, the daily egg production rate significantly decreased during weeks 6–12 compared to weeks 0–6, while the feed-to-egg ratio and body weight increased. Plant extracts can serve as natural additives in poultry farming, improving production performance, product quality, and antioxidant function. Currently, there are few reports on the application of labdane-type compounds in poultry clinical trials, but some diterpenoids have demonstrated health-regulating effects. For example, labdane diterpenoids from *Forsythia* exhibit anti-inflammatory and antiviral activities (Xiang et al., 2020), suggesting potential benefits for poultry health. Compared to other natural products such as perillol, the relatively rigid bicyclic structure and secondary alcohol group of sclareol more closely resemble the backbone of natural hormones. This suggests its pharmacological action may be more inclined toward acting as a ligand, binding to specific nuclear or membrane receptors to regulate downstream gene transcription (Tang et al., 2025). Sclareol's larger molecular size and stronger hydrophobicity confer high lipophilicity, enabling its natural accumulation in intestinal epithelial cell membranes, liver cells, and adipose tissue. This forms a natural sustained-release system, allowing it to exert prolonged effects on the gut microbiota environment and improve digestion and absorption-related functions. Based on this, we designed a longer experimental cycle to allow it to fully take effect, dividing it into two phases for comparison. As anticipated, better results were observed in the later phase of the trial. This study found that the increase in egg production rate and the reduction in cracked egg rate occurred simultaneously in the sclareol-treated groups, indicating a dual mechanism of action: on one hand, the antimicrobial properties of sclareol may regulate gut microbiota, improving calcium absorption efficiency; on the other hand, its antioxidant activity may protect the function of oviductal shell gland cells. A similar improvement in eggshell strength and plasma calcium levels was observed in laying hens supplemented with oregano thymol (Ding et al., 2017).

Serum antioxidant status was assessed using key biomarkers: total antioxidant capacity (T-AOC), superoxide dismutase (SOD), and glutathione (GSH) (Silvestrini et al., 2023). Under oxidative stress, reactive oxygen species accumulation triggers lipid peroxidation, leading to malondialdehyde (MDA) formation (Demirci-Çekiç et al., 2022), which serves as a key indicator of oxidative damage extent. (Hashemi et al., 2012) found that dietary supplementation with 7.5 g/kg *Euphorbia* extract increased serum SOD levels and reduced MDA content in broilers. In this study, supplementation with 200 and 400 mg/kg sclareol primarily increased serum T-AOC and T-SOD activities at 42 days and reduced MDA levels at 84 days (*P <* 0.05), consistent with Hashemi's findings. This suggests that sclareol is non-toxic and enhances antioxidant capacity at different stages. Notably, the reduction in serum MDA levels in the sclareol-supplemented groups (over 20 %) exceeded that reported by (Büyükkılıç Beyzi et al., 2020) for 250 mg/kg vitamin E supplementation (12 %), possibly due to sclareol's unique bicyclic diterpene structure, which effectively scavenges free radicals through hydrogen atom transfer (HAT) or sequential proton loss electron transfer (SPLET) mechanisms (Vo et al., 2020) . Similar improvements in production performance and antioxidant effects have been reported for other diterpenoids, such as andrographolide (Luo et al., 2023) and forskolin (Yang et al., 2022).

Villi are the primary sites for nutrient digestion and absorption, and increased villus height expands the absorptive surface area. The higher antioxidant enzyme activity at the villus tips compared to the crypt regions helps protect villus cells from oxidative damage (Turan, 2007). This study found that the antioxidant capacity of different intestinal segments in aging broiler breeders varied, with the duodenum exhibiting the highest antioxidant capacity, followed by the jejunum and ileum. This may be related to the gradual decrease in villus height along the intestinal tract in poultry (Yamauchi et al., 2010) . The villus height-to-crypt depth ratio (V/C) is one of the most important parameters related to nutrient absorption (Nguyen, 2021). A higher V/C ratio typically indicates stronger intestinal absorption function, as increased villus height expands the absorptive surface area (Lei et al., 2015). Additionally, changes in the V/C ratio are associated with intestinal antioxidant capacity, with a higher ratio suggesting faster intestinal cell turnover (Zhu et al., 2013), thereby maintaining antioxidant balance. The results showed that sclareol significantly increased villus height or crypt depth in all intestinal segments, with the most pronounced effects observed in the duodenum and jejunum of the medium- and high-dose groups (MSCL and HSCL). These groups also showed significant improvements in the V/C ratio, consistent with the results of the oregano thymol control group. Given the accumulation of aging-related molecules in crypt stem cells and the superior effects observed at 84 days compared to 42 days, long-term sclareol supplementation may delay intestinal epithelial aging through sustained antioxidant activity, with 200 mg/kg being the optimal dose.

The intestine, as a vital organ for nutrient digestion and absorption, plays a crucial role in maintaining overall health through its antioxidant function. The intestinal antioxidant system protects barrier integrity, prevents oxidative stress-related diseases, and promotes gut microbiota balance, thereby supporting intestinal health (Kunst et al., 2023). The results showed that sclareol significantly enhanced the antioxidant capacity of all intestinal segments at 42 and 84 days, primarily by upregulating total superoxide dismutase (T-SOD) activity and reducing malondialdehyde (MDA) levels in the duodenum and jejunum. Although the magnitude of these changes was slightly weaker than that observed in the oregano thymol control group, sclareol still demonstrated potential in improving intestinal antioxidant function. These findings align with the observed increase in villus length, suggesting that sclareol may reduce oxidative stress and tissue damage caused by lipid peroxides, restore SOD antioxidant enzyme activity, protect intestinal epithelial cells from free radical damage, promote mucosal repair, and maintain barrier integrity. The effects of sclareol were more pronounced in the duodenum and ileum, with the 400 mg/kg dose showing the best results, likely due to the physiological roles and structural characteristics of these segments. In this study, sclareol significantly increased the activity of superoxide dismutase and glutathione peroxidase in the intestinal mucosa. This signifies a substantial enhancement of the gut's local antioxidant defense capacity. This functional protection directly mitigated lipid peroxidation and membrane damage in intestinal epithelial cells caused by reactive oxygen species attack. Direct morphological evidence was observed in the treatment group, where significantly improved villus height/crypt depth ratios were noted, indicating that reduced oxidative damage helped maintain intestinal epithelial integrity and regenerative capacity.

Digestive enzymes are essential for nutrient digestion and absorption in poultry. The intestinal mucosa secretes various digestive enzymes, such as amylase, enterokinase, and lipase, which break down nutrients into absorbable molecules, enhancing the digestion of carbohydrates, proteins, and fats (Kuzmina et al., 2024). The results showed that dietary supplementation with 200–400 mg/kg sclareol primarily increased trypsin and lipase activities at 42 and 84 days, with the 400 mg/kg dose being the most effective. Numerous studies have shown that flavonoids, saponins, polysaccharides, and terpenoids in plant extracts can enhance digestive enzyme activity through different mechanisms (Niu et al., 2020). Phenolic hydroxyl or glycosylated structures may directly bind to enzyme active sites, while essential oil components (terpenoids) may promote goblet cell proliferation, increase mucin secretion, optimize the chemical barrier, and reduce the inhibitory effects of inflammatory factors on enzyme synthesis and secretion, thereby indirectly enhancing enzyme activity (Zhao et al., 2019). The lipophilic nature of terpenoids in essential oils allows them to penetrate cell membranes and modulate signaling pathways, indirectly promoting enzyme secretion. For example, thymol increases bacterial cell membrane permeability, leading to cytoplasmic leakage (Bouyahya et al., 2019), and has been shown to activate the EGFR pathway, promoting tight junction protein synthesis and improving intestinal health in poultry (Wen et al., 2025). As a sclareol containing hydroxyl and terpene structures, sclareol's lipophilic properties may contribute to its ability to enhance intestinal mucosal antioxidant enzyme activity.

Sclareol is known for its antimicrobial properties, but its effects on gut microbiota remain underexplore. This study investigated how sclareol alters cecal microbiota diversity, composition, and community structure. The LHCL group showed a significant increase in the Chao1 index, similar to the ORE group, indicating that both sclareol and oregano oil can enhance microbial richness. The MHCL group exhibited a similar trend, while the HHCL group showed a non-significant decrease, suggesting that lower doses of sclareol may be more beneficial for microbial diversity, whereas higher doses could potentially cause dysbiosis.

For β-diversity, the Jaccard index was chosen to focus on compositional changes after 12 weeks of intervention. PCoA and hierarchical clustering revealed distinct separations among groups. The LHCL and MHCL groups clustered closer to the ORE group, while the CON and HHCL groups were more distant, indicating that low and medium doses of sclareol, like oregano oil, promoted a healthier microbial structure.

At the phylum level, the Firmicutes/Bacteroidota (F/B) ratio, a marker for gut health and metabolic status^[153] [154]^., was significantly lower in the LHCL and MHCL groups. This suggests sclareol may improve metabolic health by modulating this balance. Genus-level analysis revealed significantly increased abundance of beneficial bacteria producing short-chain fatty acids (SCFAs) like butyrate (e.g., *Phocaeicola_A* and *UBA1819*) in both LHCL and MHCL groups, consistent with findings from using cottonseed meal fermentation in white-feathered broilers (Niu et al., 2020). Conversely, the HHCL group exhibited a significant reduction in Bacillus species, suggesting potential adverse effects associated with high dosages.

Analysis of differential species revealed that the CON group had higher levels of potentially detrimental genera like *Akkermansia* and *Pseudomonas-E*. The genus *Akkermansia* is a known mucin-degrading bacterium, and its increased abundance may reflect damage to the intestinal mucus layer or metabolic abnormalities (Qu et al., 2023). Certain strains of Pseudomonas-E may exhibit opportunistic pathogenicity, and their presence may be associated with intestinal inflammation or impaired barrier function (Singh et al.). These findings indicate that sclareol may protect intestinal morphology and digestive enzyme activities by promoting mucin secretion and exerting anti-inflammatory effects in the gut. The differentially abundant microbiota in the HSCL group included *Faecalibacterium, Megasphaera A*, and *Bariatricus. Faecalibacterium* is a core butyrate-producing genus. Butyrate promotes energy metabolism in intestinal epithelial cells and suppresses inflammation. Furthermore, butyrate can increase H3K9ac, thereby stimulating the PPARγ/CD36/StAR pathway to enhance ovarian steroidogenesis and activating PGC1α to improve mitochondrial dynamics and alleviate oxidative damage in the ovaries (Ye et al., 2021). This suggests that the improvement in egg-laying capacity associated with sclareol may be linked to these alterations in the gut microbiota. *Megasphaera* is involved in carbohydrate metabolism (Li et al., 2022b), and changes in its abundance may reflect enhanced energy utilization efficiency, which is consistent with the observed increase in intestinal mucosal amylase activity.

Predicted functional analysis of the cecal microbial community revealed that the chicken cecal microbiota possesses extensive metabolic capabilities, involving pathways related to biosynthesis, degradation, energy production, and detoxification. The oregano essential oil (ORE) treatment had broad effects on microbial metabolism. Compared to the CON group, the ORE group significantly upregulated 13 pathways, including eukaryotic ribosome biogenesis. These changes affected not only amino acid metabolism but also fatty acid and energy metabolism, potentially promoting processes such as cell proliferation, DNA repair, and antioxidant defense. This suggests that under ORE treatment, the gut microbiota may enhance glycolysis, pyruvate metabolism, NADPH generation, and ribose synthesis, thereby increasing energy production to support antioxidant defense or other anabolic processes. The effects of sclareol treatments (LHCL, MHCL, HHCL) on microbial functional pathways were relatively consistent across doses, primarily characterized by the upregulation of fatty acid and energy metabolism and the downregulation of ribosome biogenesis and protein synthesis. This indicates that sclareol may promote energy production and fatty acid metabolism in beneficial bacteria while inhibiting the growth and protein synthesis of harmful bacteria. The similar trends across doses suggest a potential dose-dependent effect of sclareol, although within the experimental range, its influence on cellular metabolism followed a consistent pattern. The HSCL treatment significantly affected host metabolic and physiological processes. The downregulation of pathways such as secondary bile acid biosynthesis, beta-lactam resistance, unsaturated fatty acid biosynthesis, and steroid biosynthesis may be linked to the modulation of host lipid metabolism, reduced antibiotic resistance, and alterations in cell membrane function, respectively. Conversely, the upregulation of pathways like beta-alanine metabolism, selenium compound metabolism, inulin and chlorophyll metabolism, and d-arginine and d-ornithine metabolism may enhance cellular signaling, metabolic regulation, and antioxidant defense mechanisms.

Overall, the gut microbiota results suggest that sclareol may reduce the abundance of opportunistic pathogens through direct bactericidal activity or by interfering with microbial competition. High-dose sclareol might influence the energy metabolism patterns of the gut microbiota by modulating pathways such as inositol phosphate metabolism or fatty acid synthesis (e.g., octadecanoic acid and inositol metabolism), thereby optimizing host nutrient absorption. Furthermore, the differentially abundant genera *Faecalibacterium* and *Megasphaera* in the HSCL group were positively correlated with the *phylum Firmicutes*, which itself is positively correlated with the egg production rate and feed conversion efficiency in broiler breeders. A *Firmicutes*-dominated microbiota may enhance nutrient absorption and reduce fat deposition, thereby promoting reproductive performance.

As enteral nutrition, the bioavailability of fatty acids in the body is a key factor determining their biological efficacy. SREBF-1 promotes fatty acid synthesis, while FATP4 assists in fatty acid uptake, collectively maintaining lipid homeostasis and function in the intestinal mucosa and indirectly influencing digestive enzyme activity (Li et al., 2022a). This study found that sclareol primarily upregulated SREBF-1 mRNA expression in the jejunum and ileum and FATP4 mRNA expression in the duodenum and jejunum. This suggests that sclareol may enhance fatty acid synthesis and uptake by upregulating SREBF-1 and FATP4, thereby improving intestinal lipid metabolism. The increase in α-linolenic acid (α-linolenic acid) levels in eggs, as observed in Chapter 2, may indicate elevated ω−3 fatty acid levels in the maternal body, which could activate PPARγ pathways and upregulate FATP4, influencing lipase or trypsin expression (McTavish, 2024), This aligns with the observed increase in intestinal mucosal digestive enzyme activity.

Our study also observed that 200-400 mg/kg sclareol intervention improved dysbiosis in aged poultry, including increased microbial abundance and enrichment of short-chain fatty acid-producing bacterial genera. We hypothesize that gut microbiota metabolites such as butyrate may stimulate increased activity of intestinal mucosal lipase(Zhao et al., 2021) and release more free fatty acids. Elevated FFA concentrations may trigger synergistic upregulation of FATP4(Seessle et al., 2024) and SREBF-1(Zhang et al., 2022) expression in intestinal epithelial cells, promoting fatty acid uptake and lipid re-esterification processes to generate more chylomicrons. Although short-chain fatty acid concentrations and hepatic responses were not directly measured, enhanced intestinal absorption efficiency may provide richer lipid substrates for the liver to synthesize and secrete the core precursor essential for yolk formation—very low-density lipoprotein yolk (VLDLy). In the liver, sclareol may also protect hepatic function through its anti-inflammatory properties, creating conditions for efficient synthesis of yolk precursors.

Enhanced VLDLy synthesis and transport more effectively support follicular development and yolk deposition, potentially underpinning the increase in average egg production rate and Harson units. Concurrent improvements in overall nutritional metabolism and gut health also benefit shell gland function, thereby increasing eggshell thickness and reducing breakage rates. In summary, the effects of sclareol may originate in the gut and indirectly improve production performance and hatching egg quality by enhancing lipid uptake capacity during the late laying phase. However, the hypothesis linking intestinal absorption to enhanced ovarian function requires validation through metabolomics in future studies.

## Conclusion

This study confirms that supplementing diets with 400 mg/kg of sclareol effectively enhances the production performance of Tianfu broiler breeders during the late laying phase (43-49 weeks of age). Its mechanism of action likely involves a combined effect of enhancing antioxidant capacity, improving intestinal morphology, and regulating gut microbiota balance. Sclareol modulates the intestinal microecology by enriching short-chain fatty acid-producing bacteria such as *Phocaicola*, which may represent one of its key mechanisms for promoting nutrient deposition within eggs. In summary, linalool demonstrates potential as a beneficial additive for enhancing intestinal health and hatching egg quality in poultry during the late laying phase.

## CRediT authorship contribution statement

**Gang Shu:** Writing – review & editing, Supervision, Resources, Project administration, Methodology, Investigation, Funding acquisition, Formal analysis, Conceptualization. **Binghua Zhou:** Writing – original draft, Visualization, Validation, Software, Project administration, Investigation, Data curation, Conceptualization. **Yang Wang:** Visualization, Supervision, Software, Project administration, Formal analysis, Conceptualization. **Zhengkun Wu:** Validation, Formal analysis, Conceptualization. **Haohuan Li:** Supervision, Methodology. **Funeng Xu:** Software, Funding acquisition. **Wei Zhang:** Supervision, Conceptualization. **Hualin Fu:** Resources, Methodology. **Lizi Yin:** Writing – review & editing, Resources, Investigation. **Felix Kwame Amever:** Visualization, Software, Investigation, Conceptualization. **Juchun Lin:** Project administration, Funding acquisition. **Yilei Zheng:** Resources, Investigation. **Xiaoling Zhao:** Project administration, Formal analysis, Conceptualization.

## Disclosures

We wish to submit an original article for publication in Poultry Science, titled “Efficacy of dietary supplementation with sclareol on performance, egg quality, Antioxidant Capacity and Intestinal Function of Tianfu Broiler Breeders in the Late Laying Period”.

This manuscript has not been published or presented elsewhere in part or in entirety and is not under consideration by another journal. The study design was approved by the appropriate ethics review board. We have read and understood your journal’s policies, and we believe that neither the manuscript nor the study violates any of these. There are no conflicts of interest to declare.

Thank you for your consideration. We look forward to hearing from you.
